# Whole blood lead levels are associated with radiographic and symptomatic knee osteoarthritis: a cross-sectional analysis in the Johnston County Osteoarthritis Project

**DOI:** 10.1186/ar3270

**Published:** 2011-03-01

**Authors:** Amanda E Nelson, Xiaoyan A Shi, Todd A Schwartz, Jiu-Chiuan Chen, Jordan B Renner, Kathleen L Caldwell, Charles G Helmick, Joanne M Jordan

**Affiliations:** 1 Thurston Arthritis Research Center, University of North Carolina, 3300 Thurston Building, Chapel Hill, NC 27599, USA; 2 Department of Biostatistics, Gillings School of Global Public Health, University of North Carolina, 135 Dauer Drive, Chapel Hill, NC, 27599 USA; 3 Division of Environmental Health, Department of Preventive Medicine, Keck School of Medicine, University of Southern California, 1540 Alcazar Street, Los Angeles, CA 90089, USA; 4 Department of Radiology, University of North Carolina, 101 Manning Drive, Chapel Hill, NC 27514, USA; 5 Centers for Disease Control and Prevention, 1600 Clifton Road, Atlanta, GA 30333, USA

## Abstract

**Introduction:**

Lead (Pb) is known to affect bone, and recent evidence suggests that it has effects on cartilage as well. As osteoarthritis (OA) is a highly prevalent disease affecting bone and cartilage, we undertook the present analysis to determine whether whole blood Pb levels are associated with radiographic and symptomatic OA (rOA and sxOA, respectively) of the knee.

**Methods:**

The analysis was conducted using cross-sectional data from the Johnston County Osteoarthritis Project, a rural, population-based study, including whole blood Pb levels, bilateral posteroanterior weight-bearing knee radiography and knee symptom data. rOA assessment included joint-based presence (Kellgren-Lawrence (K-L) grade 2 or higher) and severity (none, K-L grade 0 or 1; mild, K-L grade 2; moderate or severe, K-L grade 3 or 4), as well as person-based laterality (unilateral or bilateral). SxOA was deemed present (joint-based) in a knee on the basis of K-L grade 2 or higher with symptoms, with symptoms rated based on severity (0, rOA without symptoms; 1, rOA with mild symptoms; 2, rOA with moderate or severe symptoms) and in person-based analyses was either unilateral or bilateral. Generalized logit or proportional odds regression models were used to examine associations between the knee OA status variables and natural log-transformed blood Pb (ln Pb), continuously and in quartiles, controlling for age, race, sex, body mass index (BMI), smoking and alcohol drinking.

**Results:**

Those individuals with whole blood Pb data (*N *= 1,669) had a mean (±SD) age of 65.4 (±11.0) years and a mean BMI of 31.2 (±7.1) kg/m^2^, including 66.6% women and 35.4% African-Americans, with a median blood Pb level of 1.8 μg/dl (range, 0.3 to 42.0 μg/dl). In joint-based analyses, for every 1-U increase in ln Pb, the odds of prevalent knee rOA were 20% higher (aOR, 1.20; 95% CI, 1.01 to 1.44), while the odds of more severe rOA were 26% higher (aOR, 1.26; 95% CI, 1.05 to 1.50, under proportional odds). In person-based analyses, the odds of bilateral rOA were 32% higher for each 1-U increase in ln Pb (aOR, 1.32; 95% CI, 1.03 to 1.70). Similarly for knee sxOA, for each 1-U increase in ln Pb, the odds of having sxOA were 16% higher, the odds of having more severe symptoms were 17% higher and the odds of having bilateral knee symptoms were 25% higher. Similar findings were obtained with regard to ln Pb in quartiles.

**Conclusions:**

Increases in the prevalence and severity measures for both radiographically and symptomatically confirmed knee OA (although statistically significant only for rOA) were observed with increasing levels of blood Pb, suggesting that Pb may be a potentially modifiable environmental risk factor for OA.

## Introduction

Osteoarthritis (OA) is the most common form of arthritis, affecting approximately 27 million U.S. adults [1]. While some risk factors for OA of the knee are known, such as older age and obesity [2], the disease process remains poorly understood and no effective disease-modifying treatments are currently available. Regional differences in arthritis prevalence [3] are suggestive of a possible contribution of environmental factors. There is a precedent for environmental factors to cause arthritis, as in Kashin-Beck disease, an endemic arthropathy largely confined to areas of China and Tibet, which is thought to be at least in part related to selenium deficiency [4]. Mseleni joint disease in Africa and Handigodu disease in India are other arthropathies thought to have an environmental contribution [5,6]. Hereditary hemochromatosis, a genetic disorder of metabolism of iron and other heavy metals [7], is frequently associated with an arthropathy with features similar to those of OA [8,9]. Given these observations, we considered the possibility of environmental metal exposure as a novel risk factor for OA.

Lead (Pb) is ubiquitous in the environment, and although overall exposure in the United States has been on the decline [10,11], regional differences remain [12]. Approximately 95% of the total body Pb burden in adults is stored in bone and has a half-life of decades [13], which contributes as much as 65% to measured whole blood Pb levels [14]. Pb deposition has been observed in cartilage and bone in human OA [15,16] and is measurable in the synovial fluid of individuals with knee OA without a history of excessive metals exposure [17]. Pb stored in bone is released chronically into the blood pool, especially during times of increased bone turnover, such as menopause [18,19], and potentially during bone remodeling as seen in OA. This makes bone both a target tissue for Pb toxicity and a persistent endogenous source of Pb [20-24]. Even mild elevations in blood Pb levels may have health consequences, including increased mortality, as shown by recent studies based on data from the Third National Health and Nutrition Examination Survey [25] and the Study of Osteoporotic Fractures [26].

Long-term exposure to Pb may affect bone and other joint structures in humans. Pb interferes with regulatory aspects of bone cellular function and matrix synthesis, with effects on dietary calcium uptake and metabolism and conversion of vitamin D to 1,25-OH vitamin D [23,27,28]. Pb exposure affects the function of bone remodeling cells, causing impaired collagen synthesis by osteoblasts and impaired resorptive capacity of osteoclasts [28]. In a study of bone samples from individuals without known bone disease or Pb exposure, researchers identified marked accumulation of Pb specifically at the cartilage tidemark, which is the transition point between the calcified and noncalcified cartilaginous matrix [29], where clefts occur as OA develops. Zuscik and colleagues [30] reported reversion of articular chondrocytes to a more primitive phenotype upon Pb exposure, with matrix degradation and mineralization as well as chondrocyte hypertrophy. A related study revealed impaired fracture healing in Pb-exposed mice, with associated delays in endochondral maturation [31]. The affinity of Pb for joint tissues, as well as the apparent role of Pb in cartilage and bone maturation and repair, suggests a potential role for Pb in the OA disease process, in which there is an imbalance of bone and cartilage remodeling with prominent involvement of the tidemark area of the cartilaginous matrix.

The current analysis uses data from the Johnston County Osteoarthritis Project, a population-based longitudinal cohort of individuals with or without OA in rural North Carolina. Residential sources of Pb contamination remain a problem in North Carolina, and rural areas have a higher percentage of older, pre-1950 housing than urban areas [32]. In addition, pesticides containing Pb and arsenic were in widespread use in North Carolina well into the latter half of the 20th century before they were banned and before occupational protective regulations became routine. Almost 20% of our study participants have farmed for at least 1 year of their lives. These potential exposures suggest that this population may have a wider range of Pb values than other populations and therefore may be useful in exploring potential effects of Pb on OA. Using data from this well-defined cohort, we performed a cross-sectional analysis to determine whether there were associations between whole blood Pb levels and either radiographically determined OA (rOA) or symptomatically determined OA (sxOA) at the knee.

## Materials and methods

This was a cross-sectional study using data from the Johnston County Osteoarthritis Project, a population-based study of OA in rural Johnston County, NC, USA. Details of this study have been reported previously [33]. Briefly, the study participants were civilian, noninstitutionalized, African-American or Caucasian adults ages 45 years and older recruited by probability sampling in six townships beginning in 1991. The Metals Exposure Sub-Study, including whole blood Pb assessment, was designed to consist of 1,700 consecutive individuals either newly enrolled during cohort enrichment in 2003 and 2004 or returning for a second follow-up visit between 2006 and 2008. The cohort enrichment sample was enriched for younger individuals, men and African American individuals, who were lost in a higher proportion than other participants over the follow-up period from study initiation [34]. Whole blood Pb levels were obtained for a total of 1,669 individuals at the same clinic visit at which radiography was performed. This study has been approved by the institutional review boards of the Centers for Disease Control and Prevention and the University of North Carolina, and all participants provided informed consent prior to participation in the study.

### Radiographically confirmed knee OA

Participants underwent bilateral posteroanterior fixed-flexion radiography of the knees in weight-bearing as previously described [33]. All films were read by a single musculoskeletal radiologist (JBR) previously shown to have high inter- and intrarater reliability (κ = 0.86 and κ = 0.89, respectively) [35]. Knee radiographs were graded according to the Kellgren-Lawrence (K-L) classification scheme (from 0 to 4). Knee rOA (joint-based) was diagnosed if a knee had a K-L grade ≥2. The severity of rOA was considered at three levels: none (K-L grade 0 or 1), mild (K-L grade 2) or moderate or severe (K-L grade 3 or 4) for each knee. Bilateral rOA was defined as a K-L grade ≥2 in both knees, and unilateral rOA was defined as a K-L grade ≥2 in only one knee (person-based).

### Symptomatically confirmed knee OA

All participants completed symptom questionnaires. A sxOA diagnosis (joint-based) was made on the basis of the presence of rOA (K-L grade ≥2) and an affirmative answer to the question, "On MOST days do you have pain, aching or stiffness in your knees?" which was asked separately with regard to each knee. The severity of sxOA was based on the question, "Is the pain, aching or stiffness in your knees mild, moderate, or severe?" which was also asked with regard to each knee, coded according to three categories (0, rOA without symptoms, 1, rOA with mild symptoms; and 2, rOA with moderate or severe symptoms). Bilateral sxOA was defined as the conjoint occurrence of at least mild symptoms in the presence of rOA in each knee (person-based). Unilateral sxOA required at least mild symptoms in one knee with rOA and no symptoms in the other knee (regardless of rOA status).

### Blood Pb level

Whole blood was collected utilizing certified metal-free blood drawing equipment and vials and was stored at approximately 4°C [36,37] until shipment in batches of 50 to 100 to the Division of Laboratory Sciences, National Center for Environmental Health, Centers for Disease Control and Prevention (CDC), for analysis. Whole blood Pb concentrations were determined at the CDC using the PerkinElmer Inductively Coupled plasma-dynamic reaction cell-Mass Spectrometer 6100 ELAN series DRC II, ELAN DRC II ICP-MS (PerkinElmer SCIEX; Concord, ON, Canada) equipped with a Meinhard nebulizer and cyclonic spray chamber (PerkinElmer). In this multielement analytical technique, blood samples are diluted with ≥18 MΩ/cm water and with diluent containing 1% vol/vol tetramethylammonium hydroxide, 0.5% disodium ethylenediamine tetraacetate, 10% ethyl alcohol and 0.05% Triton X-100. Gold is added to reduce intrinsic mercury memory effects. Bismuth was added for the internal standardization of Pb. The samples were prepared with the following ratio: sample:water:diluent = 1:1:48. Pb was quantified on the basis of the ratio of analyte signal to that of the internal standard signal in peak hopping mode. The calibration was external matrix-matched, control samples were assessed along with participant samples and accuracy was verified by the analysis of standard reference material (SRM 955c) from the National Institute of Standards and Technology. For Pb, the limit of detection in micrograms per deciliter was based upon the standardization of base blood material and was 0.25 μg/dl (*n *= 284). The interassay precision (relative standard deviations) for lead was 3.2% at a level of 2.89 μg/dl.

### Potential confounders

Covariates included age in years, sex, self-reported race (African-American or Caucasian), body mass index (BMI) and indicators of current smoking and alcohol drinking. BMI was determined at a clinic evaluation by measuring participants' weight and height and calculating their BMI in kilograms per square meter. Current smoking status was based on the answer to the question, "Do you smoke cigarettes now?" and was dichotomized to either current smoker or current nonsmoker. Current alcohol drinking was based on the question, "Do you currently drink alcoholic beverages?" and was dichotomized to either current drinker or current nondrinker. Because of demographic differences between the two cohorts (the second follow-up sample, from 2006 to 2008, consists of older individuals, while the cohort enrichment sample, from 2003 to 2004, was enriched for younger individuals, men and African-Americans), sequential modeling, including a cohort indicator, was done to assess for any cohort effect on the models. First, a test for interaction between a cohort indicator and blood Pb level was found to be nonsignificant. Second, inclusion of the cohort indicator as a confounder did not alter effect estimates, and this term was therefore dropped from the models.

### Statistical analysis

The natural log-transformed lead was used to assist in the normalization of the distribution for blood Pb levels, since the distribution of Pb levels on the original scale was right-skewed. Truncation of ln Pb levels at the top 99.5th percentile was attempted because of a few high values, but because this did not change the results, it was not done in the final analyses. ln Pb variables were used on the continuous scale and were also categorized into quartiles. Descriptive statistics were calculated for the whole sample and for those with complete data for rOA and sxOA. One-way analysis of variance was performed to test differences in the distributions of continuous variables (for example, Pb levels) across subgroups of interest.

The proportional odds assumption was tested for three-level ordinal outcomes (laterality and severity). A proportional odds model assumes that the relationship between each independent variable of interest and the ordinal outcome is similar across the incremental measures of outcome variables. This method generates a single odds ratio to describe the comparison between the highest category and the other combined categories (for example, moderate or severe OA versus mild OA or none) and between the highest categories and the lowest category (for example, mild, moderate or severe OA versus none). If the proportional odds assumption was violated, a generalized logit model (which generates an odds ratio for the comparison between each level and the reference category) was used. The analysis was done as person-based (for the laterality outcomes) and joint-based (for the presence and severity outcomes). For joint-based analyses, each participant was scored for two observations, one for each knee, and generalized estimating equations were used to account for intraperson dependency of the data. All models were adjusted for age, sex, race, BMI, current smoking and current alcohol drinking.

Interactions between blood Pb levels and each of the covariates were tested jointly in the models. Adjusted *P *values of 0.1 or less were considered statistically significant, and for those models with significant interactions, two-way interaction terms were assessed. If these individual *P *values were significant at the 0.1 level, then appropriate subgroups were examined using stratification and adjusted odds ratios (aORs) and 95% confidence intervals (95% CIs) were calculated separately for each subgroup. All analyses were performed using SAS version 9.2 software (SAS, Inc., Cary, NC, USA).

## Results

A total of 1,669 individuals with complete blood Pb level data were available for the analysis, including 1,635 participants with complete data to define knee rOA outcomes and 1,605 with complete data for the assessment of knee sxOA outcomes. The average age of participants was 65 years, and the participants' mean BMI was 31 kg/m^2^. About two-thirds of participants were Caucasian women, of whom fewer than 25% reported current smoking or drinking (Table 1). The median blood Pb level in the sample was 1.8 μg/dl (range, 0.3 to 42.0 μg/dl). Knee rOA was present in 40% of the participants. Bilateral knee rOA was present in 23% of all participants, and 26% had moderate to severe knee rOA. Smaller numbers of participants had sxOA (present in 24%, bilateral in 11% and moderate or severe in 17%) (Table 1). Higher ln Pb levels were seen in African-Americans, men, those with lower BMI, current smokers and current alcohol drinkers (Table 2). There were no associations between ln Pb level and rOA or sxOA outcomes in these unadjusted bivariate analyses. There were no significant interactions between any of the covariates and ln Pb level for either rOA or sxOA outcomes.

**Table 1:** Characteristics of the study population^a^

Characteristic	Knee rOA (*n *= 1635)^b^	Knee sxOA (*n *= 1605)^b^
Mean whole blood Pb^c^, μg/dl (±SD)	2.4 (2.5)	2.4 (2.5)
Mean age, yr (±SD)	65.3 (11.0)	65.2 (11.0)
Mean BMI, kg/m^2 ^(±SD)	31.2 (7.1)	31.2 (7.1)
African-Americans, *n *(%)	582 (35.6%)	573 (35.7%)
Women, *n *(%)	1085 (66.4%)	1069 (66.6%)
Current smoker, *n *(%)	259 (16.5%)	257 (16.6%)
Current drinker, *n *(%)	333 (21.2%)	329 (21.2%)
Knee outcomes	rOA	sxOA
Present, *n *(%)	659 (40.3%)	384 (23.9%)
Bilateral, *n *(%)	377 (23.1%)	170 (10.6%)
Moderate to severe, *n *(%)	426 (26.1%)	271 (16.9%)

^a^rOA, radiographic osteoarthritis; sxOA, symptomatic osteoarthritis; SD, standard deviation; Pb, lead; BMI, body mass index; ^b^samples with complete data for knee rOA and knee sxOA, respectively; ^c^median, 1.8 μg/dl (range, 0.3 to 42.0 μg/dl).

**Table 2:** Mean ln-transformed Pb levels by covariate status (*n *= 1,669^a^)

Characteristics and strata	Mean ln Pb (±SD)	*P *value^b^
Race		<0.001
African-American	0.86 (0.70)	
Caucasian	0.51 (0.54)	
Sex		<0.001
Women	0.52 (0.56)	
Men	0.85 (0.68)	
BMI		<0.001
>30	0.58 (0.59)	
≤30	0.68 (0.65)	
Current smoking		0.006
No	0.57 (0.59)	
Yes	0.97 (0.67)	
Current drinking		<0.001
No	0.56 (0.58)	
Yes	0.91 (0.70)	
Knee rOA		0.1
No	0.64 (0.64)	
Yes	0.63 (0.60)	
Knee sxOA		0.1
No	0.65 (0.64)	
Yes	0.60 (0.60)	

^a^For rOA, *n *= 1,635; for sxOA, *n *= 1,605; for smoking, n = 1,605; for drinking, *n *= 1,606; ln Pb, natural log-transformed lead; SD, standard deviation; rOA, radiographic osteoarthritis; sxOA, symptomatic osteoarthritis; ^b^*P *values represent differences in mean ln Pb values between strata for each characteristic.

### Knee rOA

Table 3 contains the aORs and 95% CIs for joint-based associations between ln Pb level as a continuous variable and the presence (versus absence) of the knee OA variables, as well as the covariates in the model. The odds of having knee rOA were about 20% higher for every 1-U increase in ln Pb level. In addition, the odds of having more severe rOA were 26% higher for each 1-U increase in ln Pb level (aOR, 1.26; 95% CI, 1.05 to 1.50). Using person-based analyses, the odds of having bilateral rOA compared with no rOA were 32% higher for each 1-U increase in ln Pb level (aOR, 1.32; 95% CI, 1.03 to 1.70).

**Table 3:** Adjusted odds ratios and 95% confidence intervals for associations between knee outcomes, continuous ln Pb and covariates (joint-based analysis)^a^

	Knee rOA		Knee sxOA	
	
Variable of interest (reference category)	aOR^b^	95% CI	aOR^b^	95% CI
ln Pb (per 1-U change)	1.20	1.01 to 1.44	1.16	0.93 to 1.45
Age (per year)	1.08	1.07 to 1.09	1.05	1.03 to 1.06
BMI (per kg/m^2^)	1.12	1.10 to 1.14	1.12	1.10 to 1.14
Sex (men)	0.96	0.77 to 1.21	1.24	0.94 to 1.63
Race (Caucasian)	1.08	0.85 to 1.36	0.84	0.64 to 1.11
Smoking (No)	0.60	0.43 to 0.85	0.59	0.38 to 0.91
Drinking (No)	0.75	0.57 to 1.00	0.72	0.50 to 1.03

^a^Reference category is no rOA for rOA outcomes and rOA without symptoms for sxOA outcomes; aOR, adjusted odds ratio; CI, confidence interval; ln Pb, natural log-transformed lead; rOA, radiographic osteoarthritis; sxOA, symptomatic osteoarthritis; BMI, body mass index; ^b^adjusted for age, sex, race and ethnicity, BMI, current smoking and current drinking.

In joint-based analyses using ln Pb in quartiles (with the lowest quartile, mean Pb 0.94 ± 0.20 μg/dl, as the reference category), the highest quartile of ln Pb (mean Pb, 4.92 ± 3.93 μg/dl) was associated with a 27% increase in the odds of having knee rOA and 34% higher odds of having more severe rOA (Table 4). There was a linear trend for these associations (for presence, *P *for trend = 0.113; for severity, *P *= 0.034), as increasing quartiles of ln Pb were associated with consistently higher odds of rOA outcomes. A similar trend was also seen in person-based analyses for laterality, where the odds of having bilateral rOA increased by 15%, 19% and 46%, respectively, for the following increasing quartiles of ln Pb: Q2 aOR, 1.15; 95% CI, 0.78 to 1.70; Q3 aOR, 1.19; 95% CI, 0.80 to 1.76; and Q4 aOR, 1.46; 95% CI, 0.96 to 2.22.

**Table 4:** Adjusted odds ratios and 95% confidence intervals for associations between ln Pb in quartiles and knee outcomes (joint-based analysis)^a^

	Knee rOA			Knee sxOA		
	
Knee outcomes^b^	ln Pb quartiles^c^	aOR^d^	95% CI	ln Pb quartiles^c^	aOR^d^	95% CI
	Q2	1.09	0.81 to 1.48	Q2	1.10	0.77 to 1.56
Presence	Q3	1.18	0.89 to 1.58	Q3	1.16	0.83 to 1.62
	Q4	1.27	0.93 to 1.74	Q4	1.24	0.86 to 1.79
	Q2	1.07	0.80 to 1.43	Q2	1.06	0.75 to 1.51
Severity^e^	Q3	1.25	0.95 to 1.65	Q3	1.16	0.83 to 1.62
	Q4	1.34	0.99 to 1.81	Q4	1.23	0.85 to 1.77

^a^aOR, adjusted odds ratio; CI, confidence interval; ln Pb, natural log-transformed lead; rOA, radiographic osteoarthritis; sxOA, symptomatic osteoarthritis; Q, quartile; ^b^reference category is no rOA for rOA outcomes, no sxOA for sxOA presence and rOA with no symptoms for sxOA severity outcomes; ^c^mean Pb (±SD) in micrograms per deciliter level by quartile: Q1 (reference), 0.94 (±0.20); Q2, 1.5 (±0.15); Q3, 2.13 (±0.24); Q4, 4.92 (±3.93); ^d^adjusted for age, sex, race and ethnicity, BMI, current smoking and current drinking; ^e^proportional odds assumption met; a common OR describes the relationship between mild, moderate or severe compared to none, or between moderate or severe compared to mild or none.

### Knee sxOA

In joint-based analyses of sxOA, the odds of having sxOA were 16% higher for each 1-U increase in continuous ln Pb (Table 3), while the odds of having more severe sxOA were 17% higher (aOR, 1.17; 95% CI, 0.94 to 1.46). In person-based analyses, the odds of having bilateral sxOA increased by 25% for each 1-U increase in ln Pb (aOR, 1.25; 95% CI, 0.90 to 1.74).

For ln Pb in quartiles, the odds of having sxOA or having more severe sxOA were 23% or 24% higher for the highest ln Pb quartile compared to the lowest quartile (Table 4). Unlike the results for rOA, the apparent linear trend was not significant (for presence, *P *for trend = 0.249; for severity, *P *for trend = 0.237). A similar pattern was seen for person-based analyses of laterality, where the odds of bilateral sxOA were 45% higher in the highest quartile of ln Pb, although this was not statistically significant (Q2 aOR, 1.16; 95% CI, 0.72 to 1.88; Q3 aOR, 1.01; 95% CI, 0.60 to 1.65; Q4 aOR, 1.45; 95% CI, 0.85 to 2.47).

## Discussion

In this large community-based sample, we identified small but significant increases in the presence, laterality and severity of knee rOA with increasing levels of whole blood Pb. A similar pattern of findings was seen for sxOA, although it was not statistically significant, likely because of smaller numbers of participants with sxOA outcomes in the sample. The associations were strongest for the most severe categories (moderate or severe, or bilateral involvement). While blood Pb levels were affected by demographic factors, being higher in African-Americans than in Caucasians, higher in men than in women, higher in those with lower BMI and higher in those who reported current smoking or current alcohol drinking, the associations with knee rOA remained statistically significant after adjustment for these factors, and no significant interactions between covariates were identified. Interestingly, while lower BMI, smoking and alcohol drinking were all protective factors in the prevalent rOA analysis, and all were associated with higher Pb levels, even after adjustment for these factors, we identified a significant effect of elevated blood Pb level on the presence of rOA. This suggests to us that this ubiquitous environmental toxicant may have a role in knee OA.

The blood Pb levels seen in our study were comparable to those reported in other studies of the health effects of blood Pb levels in nonoccupationally exposed individuals [10,19,20,24,26,38-40]. These levels are far below the threshold for chelation therapy (50 μg/dl or higher with severe symptoms, or greater than 100 μg/dl with or without symptoms [41]).

The associations between rOA and whole blood Pb levels could be due to a detrimental effect of Pb on the joints leading to structural damage, or the increased Pb levels could reflect an increased rate of bone turnover in OA leading to increased release of Pb from bone. The associations between blood Pb level and sxOA could be related to modulation of pain perception by Pb itself, given its known neurotoxic effects [42]. Although we are unable to draw any causal conclusions on the basis of this cross-sectional data analysis, there is support for a potential causative effect of Pb in OA. Pb is locally toxic to bone, and intraarticular Pb, as demonstrated with retained Pb bullets, can lead to arthritis, synovitis and even systemic toxicity [43,44]. Mice exposed to Pb have delayed fracture healing and reduced endochondral maturation [31], suggesting a potential impact of Pb on bone remodeling, a process seen in OA. A study of trace elements in bone found significantly lower Pb concentrations in femoral heads of patients undergoing total hip replacement for OA than in those with hip fracture or in necropsy controls, suggesting release of Pb into the circulation from the remodeling OA bone [45]. In contrast, a study of articular cartilage and subchondral bone from individuals without bone disease or known Pb exposure showed differential specific accumulation of Pb in the tidemark region [29]. The tidemark represents the transition between calcified and uncalcified cartilage, an area known to advance, duplicate and develop clefts during the development of OA. These findings suggest that Pb may have a direct effect on the joints in OA beyond the release of Pb into the circulation as a consequence of bone remodeling. It is also possible that early changes in OA lead to the release of Pb from bone, thus aggravating joint damage. A mechanism by which Pb exposure may increase the susceptibility of osteoblasts to environmental toxins has recently been proposed [46], and it may be that regardless of causality, once the Pb levels are increased, a cycle of increased susceptibility to toxic damage may begin.

Another mechanism by which Pb may contribute to pathology in OA is through nitric oxide (NO), an important mediator of oxidative stress. Chondrocytes have long been known to express inducible nitric oxide synthase (NOS) [47], and recently a greater role for NO in the pathogenesis of OA has been recognized [48,49]. Increased production of NO and associated molecules has been noted in OA joints and specifically in chondrocytes [48]. Beneficial effects of NO on chondrocytes and the cartilage matrix, mediated through constitutive NOS, as well as negative effects mediated by inducible NOS, have been identified [48,50]. Differential effects on pain based on the pathway and local environment where NO is produced have also been found [48,51]. Interestingly, and pertinent to the current study, Pb can both increase reactive oxygen species and reduce the availability of NO, thereby increasing potential oxidative stress [52]. In studies of Pb-induced hypertension, a cycle of increased oxidative stress leading to nuclear factor-κB-mediated inflammation and apoptosis, followed by additional oxidative stress from the released inflammatory mediators and subsequent increased inflammation, has been hypothesized [52] and is thought to be aggravated by Pb exposure, which contributes to inflammation, apoptosis and reduced NO production [53]. Increased Pb levels may therefore lead to more severe rOA and more severe symptoms, as seen in the current analysis, by contributing to increased inflammation and oxidative stress mediated through a reduction in NO.

There are some limitations to the current study. Whole blood Pb measurements were used as an economical and readily available biomarker for Pb exposure [54,55]. Although specialized X-ray fluorescence techniques to assess bone Pb are considered the "gold standard" for measurement of Pb storage, this procedure is expensive and not widely available [54,55]. Since blood Pb levels reflect recent exposure as well as the mobilization of Pb from bone [19,54], and since blood Pb level has been associated with all-cause mortality, cardiovascular disease and renal disease [25,26,38-40,56], this measure of Pb was used instead. The current study is also limited because of its cross-sectional design, although the potential for future longitudinal studies of Pb in this population exist. The strengths of this study include its community-based biracial sample, large sample size and the availability of high-quality radiographic and symptomatic outcome data gathered in a standard manner.

## Conclusions

We have identified a novel association between whole blood Pb levels and the presence and severity of rOA and sxOA of the knee. These observations were seen in both men and women and in both African-Americans and Caucasians. This could represent a direct toxic effect of Pb on joint tissues, or it may represent an indirect effect of increased Pb release from bone secondary to bone remodeling. Pb may represent a novel, modifiable risk factor in patients with OA. Longitudinal studies in this and other populations will help to clarify and validate these findings.

## Abbreviations

aOR: adjusted odds ratio; BMI: body mass index; CDC: Centers for Disease Control and Prevention; CI: confidence interval; K-L: Kellgren-Lawrence; ln Pb: natural log-transformed lead; NO: nitric oxide; NOS: nitric oxide synthase; OA: osteoarthritis; Pb: lead; rOA: radiographic osteoarthritis; sxOA: symptomatic osteoarthritis.

## Competing interests

The authors declare that they have no competing interests.

## Authors' contributions

AEN participated in the design of the study and drafted the manuscript. XAS and TAS participated in the design of the study and performed the statistical analysis. JC and CGH assisted with manuscript editing and preparation. JBR read the radiographs and assisted with manuscript editing. KC performed the blood lead analyses. JMJ conceived of the study, participated in the study design and helped draft the manuscript. All authors contributed to the interpretation of the study findings and approved the final manuscript.
